# New Additions to the Mammal List Documented in the Portuguese Red Data Book

**DOI:** 10.3390/ani14172514

**Published:** 2024-08-29

**Authors:** Maria da Luz Mathias, António Mira, Joaquim Tapisso, Ricardo Pita, Tomé Neves, João Alexandre Cabral, Paulo Barros, Ana Rainho, Paulo Célio Alves, João Queirós, Joana Paupério, Marisa Ferreira, Catarina Eira, Marina Sequeira, Luísa Rodrigues

**Affiliations:** 1 Centro de Estudos do Ambiente e Mar (CESAM), Faculdade de Ciências da Universidade de Lisboa, 1749-016 Lisboa, & Universidade de Aveiro, 3810-193 Aveiro, Portugal; jstapisso@ciencias.ulisboa.pt (J.T.); catarina.eira@ua.pt (C.E.); 2 Centro de Ecologia, Evolução e Alterações Ambientais (Ce3C), Faculdade de Ciências da Universidade de Lisboa, 1749-016 Lisboa, Portugal; amrainho@ciencias.ulisboa.pt; 3 Instituto Mediterrâneo para a Agricultura, Ambiente e Desenvolvimento (MED), Universidade de Évora, Pólo da Mitra & Change, 7002-554 Évora, Portugal; amira@uevora.pt (A.M.); ricardo.pita@gmail.com (R.P.); 4 Centro de Investigação em Biodiversidade e Recursos Genéticos (CIBIO/BIOPOLIS), Universidade do Porto, Campus de Vairão, 4485-661 Vairão, Porto, & Estação Biológica de Mértola (EBM), & Instituto Superior de Agronomia, 1349-017 Lisboa, Portugal; tome_neves@hotmail.com (T.N.); pcalves@fc.up.pt (P.C.A.); joao.queiros@cibio.up.pt (J.Q.); joanapcastro@gmail.com (J.P.); 5 Centro de Investigação e Tecnologias Agroambientais e Biológicas (CITAB), Universidade de Trás-os-Montes e Alto Douro, 5001-801 Vila Real, Portugal; jcabral@utad.pt (J.A.C.); pbarros@utad.pt (P.B.); 6 European Molecular Biology Laboratory, European Bioinformatics Institute, Wellcome Genome Campus, Hinxton CB10 1SD, UK; 7 Sociedade Portuguesa de Vida Selvagem (SPVS), 3080-530 Figueira da Foz, Portugal; mctferreira@socpvs.org; 8 Instituto da Conservação da Natureza e das Florestas (ICNF), 1495-165 Algés, Portugal; marina.sequeira@icnf.pt (M.S.); luisa.rodrigues@icnf.pt (L.R.)

**Keywords:** mammals, distribution, conservation, biodiversity

## Abstract

**Simple Summary:**

Red Data Books, by encompassing comprehensive lists of species and their statuses, are crucial indicators of the health of the world’s biodiversity and powerful tools to guide conservation actions. Moreover, the adopted IUCN system for extinction risk assessment, structured around specific categories and criteria, provides an objective framework that enhances our understanding of the conservation trends of individual species over time. This study outlines the procedures used for collecting, processing, and categorizing data concerning the most recent additions to the list of mammals on mainland Portugal, as documented in the recently revised Red Book. This selection is based on three requirements: (i) species newly recorded, (ii) species newly designated taxonomically, and (iii) newly occasional species. A total of 10 species meet the requirements (i) and (ii), organized into four taxonomic groups: Eulipotyphla (1), Chiroptera (4), Rodentia (2), and Cetacea (3). All these species have been assessed for the first time, with most falling into a threatened category. Additionally, six more Cetacea species were listed as vagrant at a regional level (requirement iii) and were not considered for category assignment in the Red Data Book.

**Abstract:**

This study outlines the procedures used for collecting, processing, and categorizing data on 16 new mammal species for mainland Portugal, belonging to four taxonomic groups: Eulipotyphla (1), Chiroptera (4), Rodentia (2), and Cetacea (9). Data collection and processing encompassed field and lab work and bibliographic compilation. Data categorization involves, whenever possible, the assessment of the approximate number of mature individuals in populations, the extent of occurrence, and the area of occupancy. The approach employed led to the classification of eight out of the 16 species into an IUCN category: two non-volant small mammals and one bat species were designated as Vulnerable, requiring ongoing monitoring; one rodent and three cetaceans were assigned to Data Deficient due to insufficient available information; and a single bat species was classified as Least Concern due to the high abundance of local populations. For small mammals and bats, alterations to natural systems and climate change emerged as the most relevant threatening factors, while for cetaceans, human activities such as fishing, commercial shipping, and tourism were identified as the primary survival risks. It is recommended to maintain action programs that assist in defining strategic orientations for the implementation of conservation measures on a case-by-case basis.

## 1. Introduction

Biodiversity has experienced increased pressure from human activities in recent decades, resulting in the extinction of several species and endangering the survival of many others [1,2]. Red Data Books play a crucial role in monitoring the conservation status of species. Adhering to the criteria established by the International Union for Nature Conservation (IUCN), which considers factors such as population size, rate of decline, range area, and the extent of occurrence and fragmentation, species are classified into threat categories, allowing us to identify those at greatest risk of extinction [3,4]. This classification is crucial for guiding the implementation of appropriate measures aimed at protecting the most threatened species and their habitats. In this context, regular updates to these assessments are vital to ensuring the effectiveness of conservation strategies and actions undertaken.

Among animal groups, mammals are particularly susceptible to environmental degradation due to their high diversity, widespread presence in various ecosystems, and a variety of life strategies (e.g., [5,6]). Numerous species are currently at risk of extinction, relying on the implementation of conservation efforts to ensure their long-term survival.

In Portugal, the initial assessment of mammal species, conducted in accordance with IUCN guidelines, was undertaken in 1990 [7]. This assessment was subsequently revised in 2005 [8] and most recently in 2023 [9]. In the latest evaluation, a total of 108 species were analyzed, including the new occurrence records of 16 species not documented in the previous assessment.

The compilation of this updated mammal list was conducted as part of a research project involving diverse multidisciplinary teams. Two main approaches were employed: extensive field work throughout the country, with a focus on Natura 2000 Sites, and laboratory analysis of samples collected during the field survey. The overarching objective of this comprehensive effort, which includes a bibliographic survey of previous studies, was to reassess the conservation status of all previously classified species, understand its trend over time, and evaluate, for the first time, the conservation condition of the newly documented species on mainland Portugal.

The present study summarizes data on the collecting methods, data processing, and conservation status categorization of the 16 newly added species to the country’s list. It encompasses all gathered data on their occurrence in the country, along with populational status, whenever possible, and ecological requirements. Special emphasis is placed on identifying the most relevant environmental factors impacting species survival, thereby guiding conservation efforts to ensure their continued presence in the country. By doing this, we aim to highlight the urgent need for ongoing monitoring of their populations, especially focusing on species with limited distributions or insufficient documented information.

## 2. Materials and Methods

### 2.1. Species Sampling and Processing

We follow the taxonomic nomenclature adopted in the revised Red Data Book [9] (based on Wilson and Reeder [10], complemented with the studies of Douady et al. [11] and Prothero et al. [12]. The common names of most species align with those in the IUCN Global Mammal Red List (https://www.iucnredlist.org/) (accessed on 2 July 2024)

The 16 new species for the mainland Portugal was categorized into three groups (Table 1):

(1) **Species newly recorded in the country**—including three Chiroptera, one Rodentia, and three Cetacea species.

(2) **Species that have undergone a change in their taxonomic classification**—including one Eulipotyphla, one Chiroptera, and one Rodentia species.

(3) **Vagrant species**—including six Cetacea species.

Various methodologies, tailored to the target species, were employed for field data collection and subsequent analysis carried out within the period 2019–2021, under a research project involving diverse teams and researchers possessing multiple expertise in mammalian biology (Supplementary Materials S1–S3).

The gathered information was supplemented with bibliographic data, in particular spanning the period from the publication of the previous Red Book to the present (2005–2021), or from the first indication of the presence of the new species in the country to the present. The data compilation consisted of, e.g., published studies, project reports, and unpublished field databases from the involved teams. Occurrence data records from national natural history museum collections and online database platforms were also considered when available (e.g., Global Biodiversity Information Facility, www.gbif.org) (accessed 2 July 2024). Pre-existing data were validated before being considered for processing, following the criteria defined by Sforzi and Lapini [23]. The distribution maps for each species (excluding marine mammals) incorporate available records at a 2 × 2 km scale, as proposed by the Red List Technical Working Group [3,24].

All species documented underwent assessment based on proposed IUCN criteria designed to identify risk factors across their range, excluding those species with very limited information (Not Evaluated, NE) or with occasional occurrences in the country (Not Applicable, NA).

Whenever possible, the Extent of Occurrence of each species (EOO) was estimated, defined as the area enclosed by the shortest continuous imaginary boundary encompassing all the known sites of present occurrence, such as the Area of Occupancy (AOO), reflecting the known occupied area within the identified extent of occurrence. The EOO was calculated from the minimum convex polygon containing all the sites of occurrence so far recorded, and the AOO was determined based on the number of 2 × 2 km UTM grids with occurrences of each species. Both values indicate whether most of its individuals are found in small and relatively isolated subpopulations. Additionally, whenever possible, based on both new and previously published information, an estimated or inferred approximate population size was also calculated, indicating the potential number of individuals capable of reproduction [3,24].

### 2.2. Data Collection on Non-Volant Small Mammals

For the new data collection on non-volant small mammals (Eulipotyphla and Rodentia), a 10 × 10 km UTM square grid was superimposed on the country map for systematic sampling. A total of 115 UTM squares were selected for sampling, with the majority located within Special Areas of Conservation (SACs) designated under the Natura 2000 Network [25].

Three complementary methods were employed:

(1) collection of barn owl (*Tyto alba*) pellets for morphological identification of skulls and teeth of prey,
(2) collection of hairs for morphological identification,
(3) transects for prospecting species presence signs, including nests, tracks, and droppings.

A minimum of 50 barn owl pellets per square was deemed valid for detecting the presence of species, even those less common [25,26], with a maximum of four collecting sites per square. Prey items were identified based on Madureira [27,28], Castels and Mayo [29], Alcântara [30], Blanco [31,32], Moreno and Balbotín [33], Turón [34], and Román [35].

Sampling of hairs was carried out exclusively in squares where no barn owl resting/nests were found or where it was not possible to collect a minimum of 50 pellets. Hair traps consisted of sets of three juxtaposed PVC tubes with a diameter of 32, 50, and 90 mm and lengths ranging from 12 to 15 cm. The different tube diameters accommodated species of different sizes and enhanced trap stability in the field. Each selected location was sampled once over a minimum of three nights.

Identification of hairs relied on microscopic analysis of their morphology (marrow characterization, cross-section, and cuticular printing) using specific identification keys (e.g., [36,37,38]) and a reference collection maintained by the teams involved. To address challenges in species-level identification, droppings found in the hair tubes were also used for genetic analysis as an additional source of information. Droppings were collected in 2 mL microtubes with 96% alcohol, which were stored at −20 °C until DNA extraction and subsequent genetic identification [39,40] (details below).

In each square, three transects, each ca. 600 m long, were conducted to detect the presence signs of small mammals (nests, tracks, and droppings). In cases where identifying the species based on the field signs was not feasible, droppings were collected, as mentioned earlier, for subsequent genetic identification.

### 2.3. Data Collection on Bats

A total of 180 sampling points were selected for bat data collection across a 10 × 10 km UTM square grid superimposed on the country map, with most of them located within Natura 2000 Sites (SACs). A total of 623 UTM squares were sampled [41]. The number of sampling points within 63 SACs varied between one and seven.

Here, two main complementary methods were employed:

(1) live captures,
(2) acoustic recordings.

In each of the sampling points, a capture station was strategically placed in the most suitable location (e.g., water points, paths, water lines), using mist nets of varying length (3 to 18 m). At each point, the nets remained open during the first 3–4 h of the night, the peak period of bat activity. Regular inspections, at intervals no greater than 15 min, were conducted throughout the sampling period [14,15]. Captures were made exclusively on nights with favorable weather conditions for bat activity (warm or mild nights with minimal wind and no rain). A single sampling period was carried out at each point. In instances where the morphological identification of captured individuals proved inconclusive, a tissue sample was collected from each wing membrane or uropatagium using a 3 mm punch for subsequent genetic analysis as referred to above (details below).

Live captures were complemented with ultrasound sampling, following a scheme involving three fixed listening points equipped with automatic ultrasound recording stations (AudioMoth—v1.1.0, Open Acoustic Devices). One station was placed at the capture site, and the other two were approximately 1 km away, resulting in a total of 540 acoustic sampling points. Acoustic sampling followed a schedule similar to that of mist netting. Recorded files without bat activity were automatically excluded using Kaleidoscope Pro v.4.5.5 software (Wildlife Acoustics Inc., Maynard, MA, USA). After removing noise data, species identification was performed visually by comparing the recordings with the identification key for bat vocalizations in mainland Portugal [42] and other references [43,44,45,46]. Previous recordings by the involved teams were also used for comparison.

The data compilation for bats also included Roosts Monitoring as a collection method, which provided relevant information on the occurrence and population size estimation of cave-dwelling species [47].

### 2.4. Data Collection on Cetaceans

The data on cetaceans occurring in mainland Portugal were mostly gathered through bibliographic searches and reports from projects, some of which involved team members or co-workers. This approach was employed to obtain information on the distribution and occurrence of the different species. Following this, the collected information underwent analysis to identify the threats and pressures faced by cetaceans in the waters off mainland Portugal [48].

For insights into strandings, two national databases were also consulted: the National Cetacean Databases (Institute for Nature Conservation and Forests, ICNF) and the Database of the Northern Strandings Networks (Portuguese Wildlife Society and University of Aveiro). The stranding data were scrutinized to extract information pertaining to various aspects of the biology of different species, such as the presence of calves or pregnant females. This information could prove valuable in drawing inferences about the nature of occurrences involving these species. For visual detections, relevant data were compiled from Maughan and Arnold [49] and Correia [50].

Whenever data are available, a reference to the occurrence of cetacean species off the Portuguese islands of Azores and Madeira is also included in the main text (e.g., [51]).

### 2.5. Genetic Analyses

The genetic identification of small mammals (from droppings samples) and bat specimens (from tissue samples) involved the following steps:

(1) DNA extraction,
(2) amplification of mitochondrial sequence fragments,
(3) sequencing of the amplified fragments by the *Sanger* or *High-throughput sequencing* (*MiSeq*) methods,
(4) comparative similarity sequence analysis for species identification.

For bat specimens, a fragment of the cytochrome oxidase I (COI) gene was amplified for each sample using the BF2 and BR2 primers [52] and a T100 thermal cycler (Bio-Rad, Hercules, CA, USA). Sequencing was performed on the *Miseq* platform, and the obtained sequences were subsequently processed bioinformatically using the OBITools program [53], following the methodology described in Galhardo et al. [54]. The specific identification of the samples was carried out using a similarity search tool for comparison with biological sequences listed in the NCBI *Nucleotide BLAST* database and in the team database [55].

For small mammals, fragments of the cytochrome-b (cyt-b) or 12S gene were amplified using the CB2F and CB3H primers for sequencing a smaller number of samples [56,57] via the *Sanger* method or the 12S-V5.1F and 12S-V5.1AND2R primers [58] for sequencing a larger number of samples using the *Miseq* platform. Sequences obtained through the *Sanger* method underwent processing using Geneious Pro 10.2.6 (Biomatters, Auckland, New Zealand) and were identified by comparison with sequences in the NCBI *Nucleotide BLAST* and *Barcode of Life* databases as well as the team database. The sequences obtained via the *Miseq* method were processed using the OBITools program and compared to those listed in the NCBI-*Nucleotide BLAST* database and the team database following a methodology similar to that applied to bat samples [55].

## 3. Results

### 3.1. Non-Volant Small Mammals

Barn owl pellets were collected in 88 squares, and 46 out of the total selected squares had hair traps placed, totaling 460 sets of PVC tubes. The survey covered a total of 300 transects, corresponding to a cumulative distance of 180 km [25].

Approximately 6000 pellets were collected, out of which 5900 underwent analysis. It was possible to identify 16,987 skulls down to the species level. In addition, 383 hair samples were collected, 2374 presence signs were recorded, and genetic analysis was performed on 238 droppings collected along transects and in hair traps.

**Figure 1:**
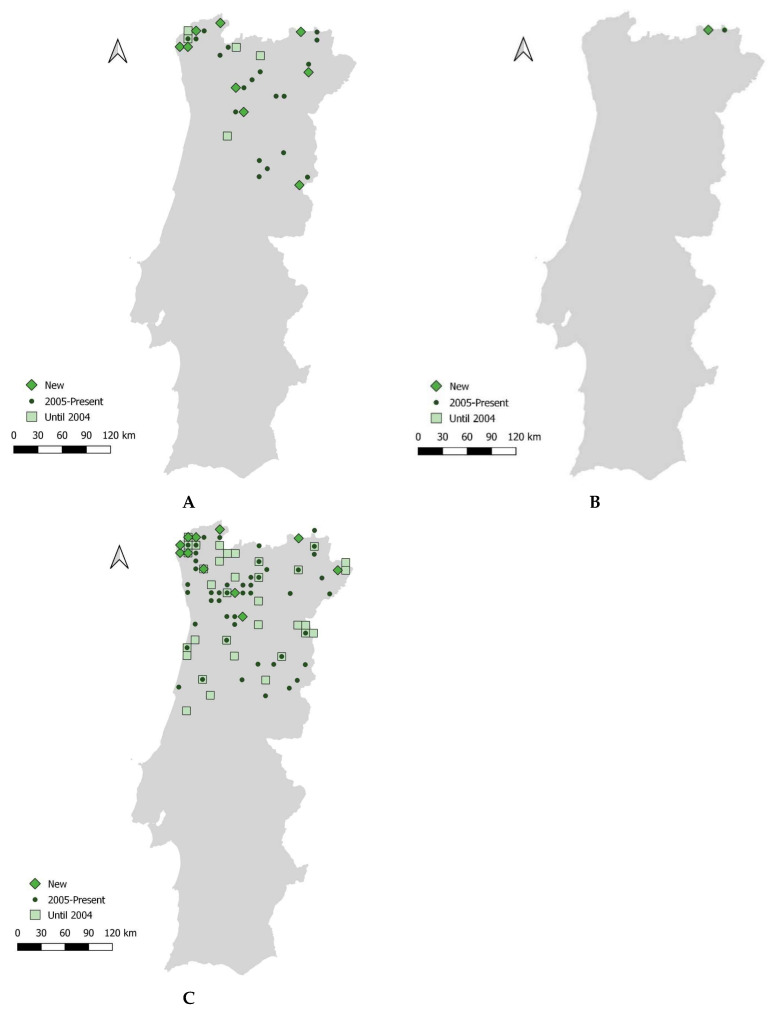
Confirmed occurrence of recently identified small mammal species in mainland Portugal: (**A**) *Neomys anomalus*, (**B**) *Chionomys nivalis*, and (**C**) *Microtus rozianus*.

The data acquired confirmed the presence of the new species *Chionomys nivalis* and the newly taxonomically designated species *Neomys anomalus* and *Microtus rozianus* in several of the selected studied sites [25] (Table 2 and Table 3, Figure 1, Supplementary Materials S2).

#### 3.1.1. *Neomys anomalus* (Cabrera, 1907) Water Shrew

This new specific entity, previously occurring in the country as *N.a. anomalus*, is now endemic to Iberia following the taxonomic revision by Igea et al. [19], uncovering a significant genetic differentiation of the Iberian populations compared to other European populations. In this study, a total of 94 records were collected, with one obtained through pellet analysis and 93 through presence signs. These included the collection of 40 droppings, of which 30 were genetically confirmed [55]. In total, records confirming the presence of *N. anomalus* were obtained in 12 different locations, encompassing 10 Natura 2000 Sites, all situated in the northernmost part of the country. Forest and agroforestry areas, crossed by small water courses, dominated the landscape in the species’ presence areas [25,59]. When comparing the results with previously recorded distribution data, the findings have extended the previously known range of this species, which corresponds to the northern third of the country (e.g., [9,60]). Considering all the collected data, both new and published, prior to the taxonomic revision, the Extent of Occurrence was calculated to be 21,158 km^2^, with an Area of Occupancy ranging between 144 and 152 km^2^. The estimated population size was 28,800 mature individuals [25].

#### 3.1.2. *Chionomys nivalis* (Martins, 1842) European Snow Vole

The European Snow Vole was first documented in the most northeastern part of the country in 2016, when two individuals, an adult male and a juvenile female, were captured in an area dominated by shrubs and low herbaceous vegetation at an altitude of 1370 m. In the present study, despite extensive fieldwork, only field signs indicative of species presence were recorded close to the location and in a similar habitat type where they had been initially collected [16,25]. Genetic analysis of droppings collected from this location did not contribute to an increase in the number of records. This suggests that the species appears to be confined to a restricted area and remains exceedingly rare in the country, with an unknown population size. Accordingly, the EOO was estimated to be 12 km^2^, with an AOO of 4 km^2^ [25]. Continuous monitoring of the species in the country is essential.

#### 3.1.3. *Microtus rozianus* (Bocage, 1865) Portuguese Field Vole

The Portuguese populations of the Portuguese Field Vole, previously considered within the species *M. agrestis*, constitute nowadays, together with the populations of northern Spain, a case of endemism in the Iberian Peninsula, presenting a high genetic differentiation and a certain degree of ecological divergence from the other species of the *agrestis* complex [20,61,62]. Here we have identified *M. rozianus* in 161 pellets, five hair samples, and 139 traces, which included 40 droppings, totaling 305 records. Genetic analysis of the droppings confirmed the presence of the species in 28 samples. These records were scattered across 15 different locations, predominantly associated with agricultural and woodland areas, with 12 locations within Natura 2000 Sites. The presence of the species was confirmed to be confined to the north and center of Portugal [63]. The gathered data, including bibliographic compilation, enabled the calculation of the EOO to be 33,189.60 km^2^ and the AOO to range between 328 and 332 km^2^ [9]. The population size was estimated to range from 16,500 to 51,000 mature individuals [25]. These estimates are somewhat consistent with the calculated effective population size of 10,000 individuals based on molecular data [20].

### 3.2. Bats

During the 180 capture sessions, a net area of 37,325 m^2^ was used, averaging 206 m^2^ net/point/hour. The average sampling duration per point was 4 h and 11 min, making a cumulative sampling effort of 753 h and 41 min. In terms of acoustic detection, a total of 540 sessions were carried out, resulting in the recording of 805,203 files spanning 2589 h and 57 min. This averages out to approximately 4 h and 49 min per sampling point [41]. A total of 448 tissue samples obtained from captured individuals were genetically analyzed [55].

**Figure 2:**
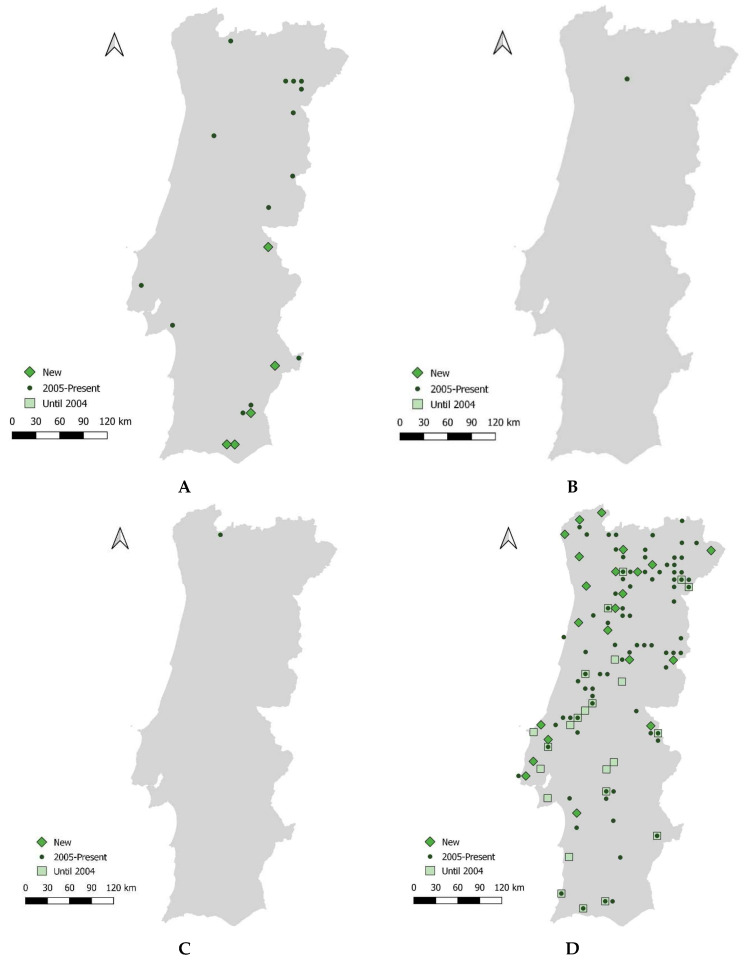
Confirmed occurrence of recently identified bat species in mainland Portugal: (**A**) *Eptesicus isabellinus*, (**B**) *Myotis crypticus*, (**C**) *Myotis alcathoe*, and (**D**) *Myotis escalerai*.

The capture sessions yielded a total of 966 individuals of different bat species, including the new species *Eptesicus isabellinus* and the newly taxonomically designated *Myotis escalerai* [41]. However, no successful captures were made for the newly documented *M. crypticus* and *M. alcathoe.* The acoustic data confirm the presence of *M. escalerai* (Table 2 and Table 3, Figure 2, Supplementary Materials S2).

#### 3.2.1. *Eptesicus isabellinus* (Temminck, 1840) Isabelline Serotine Bat

This species, initially considered a subspecies of *Eptesicus serotinus*, was recently confirmed as distinct [64]. Due to the morphological similarity between *E. serotinus* and *E. isabellinus*, only genetically confirmed records were assigned to either species.

In the present study, only 12 individuals were captured, out of which the identification of 10 individuals was confirmed by genetic analysis [41,55]. The presence of this species has been confirmed in only five surveyed locations, all situated within SACs. The percentage of occurrence in captures was 3.33%. The Isabelline Serotine inhabits natural shelters such as rock crevices and occasionally tree cavities and human structures [65]. Here, the species was captured in habitats dominated by native forest, shrubland, and agricultural areas. The obtained information has expanded its geographic range, identifying a predominant presence in the southern region and along the eastern half of the country. The gathered data, including bibliographic compilation (e.g., [9,47]), enabled the calculation of the EOO to be over 20,000 km^2^. On the other hand, the AOO of *E. isabellinus* was under 500 km^2^, but the population size was estimated to exceed 10,000 individuals [66].

#### 3.2.2. *Myotis escalerai* Cabrera, 1904, Iberian Natterer’s Bat

The Iberian Natterer´s Bat was, until recently, synonymized as *M.nattereri* [67], but it is currently recognized as a valid species [68]. All the previous data regarding *M.nattereri* in the country has been validated as *M. escalerai*.

Among the captured individuals, *M. escalerai* stood out as one of the most frequently encountered species, with 86 individuals, 14 of which were genetically confirmed. In total, the data collected for *M. escalerai* corresponds to 88 records (86 captures and 2 acoustic detections), encompassing 22 different locations, including 15 SACs. The percentage of occurrence in captures was 16.67%. *M. escalerai* is a strict cave-dwelling species. Obtained records correspond to areas dominated by resinous forest, shrubland, and forest patches dominated by exotic plants. Findings indicate an enlargement of its previously known geographic distribution, with a higher prevalence in the northern regions of the country. The EOO of this species was estimated to be over 10,000 km^2^, while the AOO was estimated to be less than 2000 km^2^ (e.g., [9]). The population size was calculated to range between 2500 and 9999 individuals, primarily based on previously gathered data from roosts monitoring [47,69].

#### 3.2.3. *Myotis crypticus* Ruedi, Ibanez, Salicini, Juste, and Puechmaille, 2019 Cryptic Myotis and *Myotis alcathoe* von Helversen and Heller, 2001 Alcathoe Whiskered Bat

Current findings indicate that the available information on both species remains extremely limited. As they are confined to highly restricted locations, no estimations could be made regarding either their Extent of Occurrence or the Area of Occupancy. Because no new occurrence records were obtained, the only data currently available on these species are those reported by Gallego et al. [14] and Rebelo et al. [15], respectively. These findings refer to very localized and diminutive records. Both species are associated with forested areas.

### 3.3. Cetaceans

**Figure 3:**
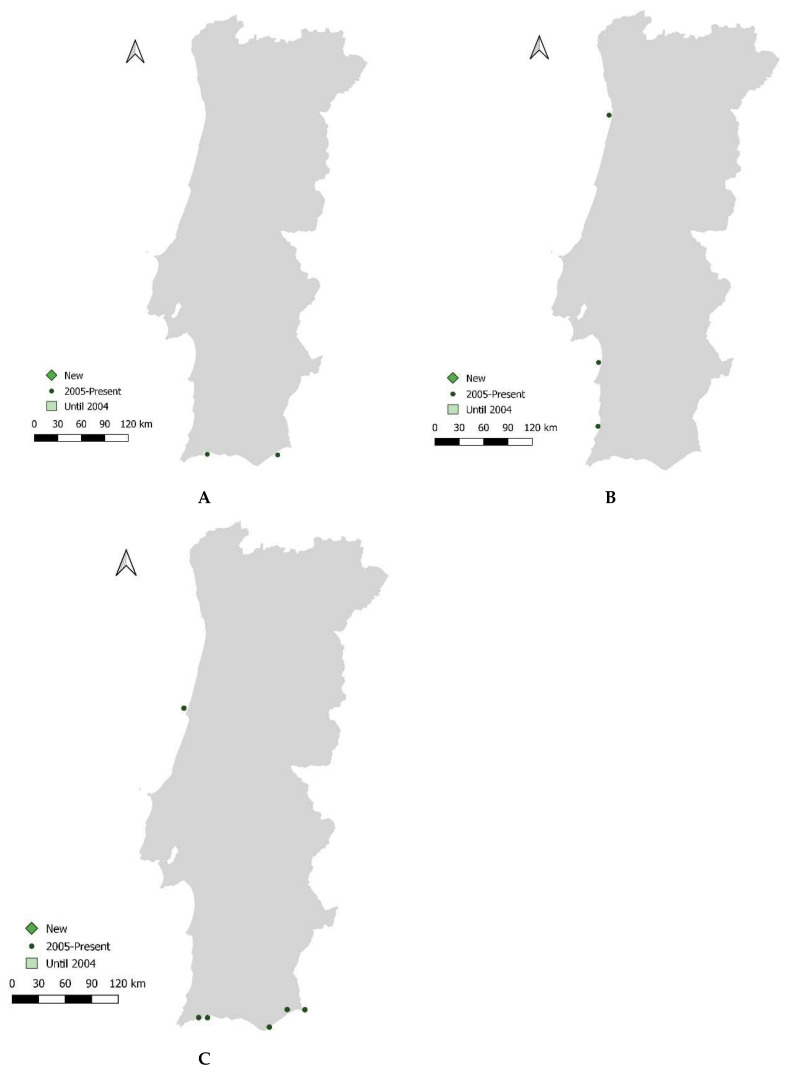
Records of strandings of (**A**) *Stenella frontalis*, (**B**) *Mesoplodon bidens* and (**C**) *M. mirus*.

Data compilation confirmed the presence of three new cetacean species (*Stenella frontalis*, *Mesoplodon bidens*, and *M. mirus*) and documented six species as vagrants in the waters off mainland Portugal. Data analysis provided insights into the frequency of strandings and sightings for each species, though most of the gathered information is limited. Strandings involved deceased and live animals, and visual detections typically occurred in areas far from the coast (Table 4, Figure 3). More detailed information is in Supplementary Materials S3.

#### 3.3.1. *Stenella frontalis* (Meyen, 1833) Atlantic Spotted Dolphin

This species stands out as the most frequently documented species, with a substantial amount of information mostly pertaining to visual detections, suggesting a potentially large population occurring in national waters [21,70]. There is also information about the stranding of two live animals, which were released back into the open sea. Atlantic Spotted Dolphins inhabit the mesopelagic layer of the ocean and are proficient swimmers [71]. Due to their oceanic nature, accurately estimating population sizes is challenging. In mainland Portugal, an estimated density of 0.023 individuals per km^2^ (CV = 0.90) was obtained in 2011 during an offshore survey conducted between 50 and 200 nautical miles from the coast [21]. A decrease in their occurrence was reported in depths below 2000 m off the coast of the Azores and Madeira [70]. The information collected has enabled the delineation of its potential occurrence area in Portuguese waters, which corresponds to waters beyond the continental platform at a considerable distance from the coast [72]. Sightings in Portuguese waters peaked at approximately 300 km from the seamounts, at a sea surface temperature of around 23 °C, and were influenced by the conditions of the sea [70]. Some visual detections of *S. frontalis* occurred inside Special Areas of Conservation for cetaceans.

#### 3.3.2. *Mesoplodon bidens* (Sowerby, 1804) Sowerby’s Beaked Whale

Data currently available on this species is limited. There is no species-specific abundance data or information on population trends in Portugal or even throughout its global range [73,74,75]. Sowerby’s Beaked Whales are seldom sighted, contributing to the lack of comprehensive data. The distribution in Portuguese waters is unknown. The only available records come from recent strandings, including that of a live calf [18,76]. The global distribution includes the Azores and the Madeira Islands [77,78].

#### 3.3.3. *Mesoplodon mirus* (True, 1913) True’s Beaked Whale

There are no known estimates of the abundance or population trends of this species in Portugal. The distribution in Portuguese waters is presently unknown [79]. The sole existing records are derived entirely from recent strandings on mainland Portugal and in the Azores, encompassing both deceased and live individuals [17,18,80]. Further research is essential to better understand the environmental factors regulating the presence of the species in Portuguese waters. The same applies to the conspecific *M. bidens*.

#### 3.3.4. Vagrant Species

Data gathered for the six vagrant species—*Balaenoptera edeni* (Lesson, 1828) (Bryde’s whale), *Globicephala macrorhynchus* (Gray, 1846) (Short-finned Pilot Whale), *Lagenodelphis hosei* (Fraser, 1956) (Fraser’s Dolphin), *Lagenorhynchus acutus* (Gray, 1828) (Atlantic White-sided Dolphin), *Lagenorhynchus albirostris* (Gray, 1846) (White-beaked Dolphin), *Kogia sima* (Owen, 1866) (Dwarf Sperm Whale)—is very scarce. The existing information does not allow for drawing any definitive conclusions regarding the presence of these species in mainland Portuguese waters. The reported data only suggest sporadic incursions into national waters [81,82,83,84,85,86].

### 3.4. Categorization and Threats

#### 3.4.1. Species Recorded for the First Time in the Country

Among the newly recorded and evaluated species, only one bat species, *Eptesicus isabellinus*, has been classified as Least Concern (LC) [66], with most of the remaining being assigned to threatened categories (Table 5 and Table 6).

The species *E. isabellinus* is locally abundant throughout its range, although the population trend is unknown. This classification aligns with the global assessment of the species [87]. No specific threats have been identified for this species, but the frequent use of human infrastructure in urban areas suggests that the destruction of shelters in buildings could pose a potential threat to the persistence of colonies [66].

The available information on the two other new bat species, *Myotis crypticus* and *M. alcathoe*, is too limited to allow their categorization (Not Evaluated NE) [88,89]. Specific threats to the establishment and maintenance of stable populations in the country have not been identified. Globally, *M. alcathoe* has been assessed as Data-Deficient [90] and *M. crypticus* as Near Threatened (NT) [91]. For both species, considering their association with forested areas, habitat destruction and eventual wildfires could significantly threaten their presence, either throughout their global range or in Portugal.

Regarding the rodent *Chionomys nivalis*, the capture of a young female has raised suspicion of a breeding population in the country [16], and despite its highly restricted location, the species was classified as Data Deficient (DD) [92]. Although populations are considered stable globally [93], predictive models based on climate change scenarios suggest a reduction in the species potential range of 32 to 78% between 2020 and 2050 [94]. Thus, it is possible to anticipate the significant impact of climate change and its consequences on habitat quality for species on mainland Portugal, particularly considering its marginal location within the global species range.

The three new cetacean species in the country—*Stenella frontalis*, *Mesoplodon bidens*, and *M. mirus*—have also been classified as Data-Deficient. Information regarding their Area of Occupancy, total population size, and trend remains unknown. The primary threats identified for *Stenella frontalis* include entanglement in fishing nets coupled with environmental disturbance. This species is one of the main target species for maritime-tourism companies in cetacean observation. On the Island of Madeira, it is one of the most frequently sighted species, accounting for 23% of all cetacean sightings [51]. *S. frontalis* is globally categorized as Least Concern [71]. The identification of *M. mirus* and *M. bidens* on mainland Portugal relies on the stranding of both deceased and live animals. For *M. bidens*, the stranding of a live newborn calf may suggest that this species uses Portuguese mainland waters for reproduction. The precise cause of these occurrences and potential threats in these waters remain unknown. Both species are categorized globally as Least Concern [73,95]. However, entanglement in fishing nets has been documented worldwide, along with underwater anthropogenic noise, as potential causes of stranding and death [96,97].

#### 3.4.2. Species That Have Undergone a Change in Their Taxonomy

The three species evaluated for the first time following a taxonomic revision—*Neomys anomalus*, *Myotis escalerai*, and *Microtus rozianus*—have all been classified as Vulnerable (VU). The restriction of their ranges following their designation as new species elevates the potential significance of threats to their populations (Table 5 and Table 6).

The categorization of *N. anomalus* is primarily due to the suspected decline in habitat quality in the years to come and the continuous reduction in population size. These declines are expected as the availability of preferred habitats in this country decreases [63,98]. The ecological dependency on riparian habitats makes the species highly susceptible to changes involving alterations in the quality of watercourses, such as riverbank destruction, dam construction, and pollution. Habitat availability may also be linked to climate change. According to proposed scenarios, the species populations may gradually decline across its global range, leading to its disappearance from the country as soon as 2041–2060 in the worst-case scenario, leaving the species range confined to the northernmost part of Iberia [60]. No global species assessment is available.

The categorization of *M. escalerai* took into account the high fragmentation of occurrences, the reduced Area of Occupancy, and the small size of populations across the country. Although the population trend is unknown, as an insectivorous species, it is anticipated that the increasing frequency of dry periods and the use of pesticides in agricultural fields may negatively impact its preferred prey, leading to a decrease in its Area of Occupancy [69]. Globally, *M. escalerai* is classified as Near Threatened, with populations suspected to be decreasing across its entire range. Roost loss, degradation, and disturbance are reported as the main threats to species survival, primarily resulting from human activities [67].

Regarding *M. rozianus*, its classification was based on the suspected reduction of the population size by more than 30% over the last 10 years, leading to a decline in the Area of Occupancy [63]. Considering that the species´ range corresponds to the northernmost part of the country, which is the area most dramatically impacted by wildfires in the country, it is also suggested that climate change, in connection with habitat degradation, may pose significant threats to populations [25]. No global species assessment is available considering its new taxonomic status.

#### 3.4.3. Occasional Species

Six species of cetaceans were sporadically observed on mainland Portugal; therefore, they were not evaluated (Not Applicable NA): *Balaenoptera edeni*, *Globicephala macrorhynchus*, *Lagenodelphis hosei*, *Lagenorhynchus acutus*, *L. albirostris*, and *Kogia sima* [81,82,83,84,85,86]. The species *Lagenodelphis hosei* and *Kogia sima* are also recorded off the islands of Madeira and the Azores [99,100]. Globally all these species were categorized as Least Concern [101,102,103,104]. However, in Europe, *Balaenoptera edeni* is categorized as Vulnerable [105], and *Kogia sima* is considered Data-Deficient [106]. Human activities such as fishing, pollution, and climate change are referred to as potential threats to their populations, with varying degrees of impact depending on the location.

**Table 6:** Classification of the main relevant threats to the new species in the country (G-global, PT-Portugal, light gray-potential threat, dark gray-identified threat).

New Species	Classification of Threats *													
	Urban Development		Agriculture		Use Biological Resources		Human Disturbance		Natural Syst. Modification		Pollution		Climate Change	
	G	PT	G	PT	G	PT	G	PT	G	PT	G	PT	G	PT
Eulipotyphla														
*Neomys anomalus*														
Chiroptera														
*Eptesicus isabellinus*														
*Myotis crypticus*														
*Myotis alcathoe*														
*Myotis escalerai*														
Rodentia														
*Chionomys nivalis*														
*Microtus rozianus*														
Cetacea														
*Stenella frontalis*														
*Mesoplodon mirus*														
*Mesoplodon bidens*														

* adapted from [107,108].

## 4. Discussion

There is nowadays heightened concern regarding the global decline in Biodiversity. According to the IUCN, mammals constitute 26% of the global species threatened with extinction. Among the main direct drivers hampering species survival are changing uses of sea and land, climate change, and pollution (see IPBES 2019 [109]).

Here we describe the data gathered for 16 new mammal species on mainland Portugal, confirmed by several teams involved in a thorough survey across the country and through bibliographic compilation. This represents approximately an 18% increase in the mammal species reported for the country since the last assessment [8], which cannot be solely ascribed to a higher number of teams and dedicated efforts investigating mammals. Rather, it can also be attributed to the adoption of more advanced tools for species identification, with an emphasis on molecular tools. Thus, the updated approaches followed also allowed the categorization of these species according to IUCN guidelines, with eight out of the 16 species being classified into an IUCN category, as listed in the recently published Red Data Book [9]: three non-volant small mammals, four bats, and three cetaceans. The remaining eight species, two bats and six cetaceans, were Not Evaluated (NE) or considered occasional occurrences in the country (NA), respectively.

For non-volant small mammals and bats, the modification of natural habitats and climate change are the most relevant and confirmed threatening factors in the national territory. Specifically, species reliant on freshwater and riparian habitats, such as Water Shrews, using these habitats for feeding and cover, or Alcathoe Whiskered Bats, known to forage above water, may be particularly sensitive to alterations stemming from the overexploitation of water resources, changes in riverbanks, dam construction, or chemical pollution [25,41]. On the other hand, the trend towards an increase in the duration and severity of drought phenomena, underlying a higher frequency of wildfires [110], along with agricultural expansion, intensification, and overgrazing, is also anticipated to pose potential threats to land-dwelling species, such as the Portuguese Field Vole and the Iberian Natterer´s Bat, which use forestry areas as feeding grounds [25,41]. These two species, along with the Water Shrew, which have recently had their taxonomic ranks changed, have been categorized as Vulnerable. The consequent expected reduction in their global range for both the Portuguese Field Vole and the Water Shrew makes their populations more susceptible to environmental changes. In addition, there is insufficient information available to assess the risk of extinction of the Alcathoe Whiskered Bat, the Cryptic Myotis, or the Snow Vole. The lack of knowledge about these rare species in the country can only be addressed through further monitoring and targeted studies focusing on factors such as distribution, population trends, and habitat requirements.

The above findings suggest that conservation measures promoting the maintenance of stable populations in preferred species’ habitats should be implemented, ideally within the framework of national and international legislation. It is noteworthy that, among mammals, rodents are the least represented group in global and regional biodiversity conservation agendas [111]. In Portugal, matching the global situation, only 20% of the rodent species in the country are subject to a legal protection regime, considering the Directives and Conventions ratified by Portugal. This bias may result in an increased risk of extinction for many rodent species in the years to come [25,112]. In contrast, all bat species in Portugal are protected by law, with the new bat species listed in Annex B-IV of the Habitat Directive. Additionally, within the scope of the Convention on the Conservation of Migratory Species of Wild Animals and Portugal’s subscription to the Agreement on the Conservation of Populations of European Bats (EUROBAT), the new four species have been included in Annex II of the Bonn Convention since 1995 [9].

Regarding cetaceans, their high mobility, often spanning large distances for feeding or migrating between breeding and feeding grounds, makes them highly susceptible to human-induced activities such as fishing, commercial shipping, and tourism. According to Braulik et al. [113], the global intensification of fishing activities is the primary cause behind the increased extinction risk faced by many cetaceans, of which approximately 300,000 are estimated by the International Whaling Commission to die accidentally in fishing nets each year.

In Portuguese waters, the main identified or potential threats to cetacean species include, besides incidental by-catch, the presence of floating marine litter and chemical and noise pollution (e.g., [114,115,116,117]). All these factors are estimated to have a negative impact on the species occurring in the country, including the newly identified species, such as the Atlantic Spotted Dolphin, the Sowerby’s Beaked Whale, and the True´s Beaked Whale. These species have been categorized as Data-Deficient, a classification that applies to nearly 30% of the cetacean species occurring in the country [9]. This value surpasses almost threefold the global value of cetaceans, still needing further data for the assessment of their conservation status [113]. The six occasional cetacean species in the country have a known global range that, until recently, did not include the waters of mainland Portugal (e.g., species from high latitudes and those from tropical and subtropical waters). However, the limited records of their occurrence indicate a marginal and sporadic presence in Portuguese waters compared to global populations. All these facts highlight the imperative of deepening our knowledge of the lesser-known cetacean species in the country. Attention should be drawn to the possibility that the risk of extinction for some of these species could worsen in the near future.

In Portugal, the protection of cetaceans is governed by several laws encompassing the prohibition or control of various activities such as deliberate capture, transportation, killing, whale-watching, or commercial fishing. Scientific observation or acoustic recording are also legally regulated. Within the Natura 2000 Network, three sites closer to the coast have been established for the protection of cetacean species. All cetacean species are included in Annex B-IV of the Habitat Directive [9].

Given the rapid pace of environmental and climate change, along with other significant threats, a monitoring program focused on both terrestrial and marine mammals is advisable to be implemented in the country. This initiative should be tailored to the specific characteristics of each species, aimed at collecting timely data on occurrence trends and the conservation status of populations. Such information would assist in defining orientations and decision-making actions by identifying the most relevant conservation measures on a case-by-case basis.

## 5. Conclusions

The present study reports the main findings of a research project aimed at assessing the conservation status of 16 newly documented mammal species on mainland Portugal, adopting the IUCN species extinction risk assessment system.

A total of 500 new occurrence records were obtained for the selected non-volant small mammal (400) and bat (100) species, along with a substantial number of compiled records from various sources, including 73 occurrence records for marine mammals. The data revealed an extension of the previously known geographic range for the Water Shrew, the Isabelline Serotine Bat, and the Iberian Natterer´s Bat. It also confirmed the rare occurrence of the Cryptic Myotis and the Alcathoe Whiskered Bat and highlighted the need for continuous monitoring of the European Snow Vole, the Atlantic Spotted Dolphin, the Sowerby’s Beaked Whale, and the True’s Beaked Whale. Furthermore, the sporadic use of continental waters by six cetacean species was reported. The identification of the main threats at a national level was compared to the globally assessed survival risks for each species.

A periodical re-evaluation of species conservation status is recommended by combining targeted sampling designs with ecological monitoring and genetic analysis. This approach will accurately assess changes in range and population trends, identify alterations in species’ ecological requirements, and, if necessary, support the implementation of species-specific conservation plans.

## Figures and Tables

**Table 1:** Recent additions to the Portuguese mammal fauna.

Order/Family	Species	First Reference in the Country
**Species recorded for the first time in the country**		
Chiroptera/Vespertilionidae	*Eptesicus isabellinus*	ICNF [13]
Chiroptera/Vespertilionidae	*Myotis crypticus*	Gallego et al. [14]
Chiroptera/Vespertilionidae	*Myotis alcathoe*	Rebelo et al. [15]
Rodentia/Cricetidae	*Chionomys nivalis*	Barros et al. [16]
Cetacea/Delphinidae	*Stenella frontalis*	Ferreira et al. [17]
Cetacea/Ziphiidae	*Mesoplodon bidens*	Ferreira et al. [18]
Cetacea/Ziphiidae	*Mesoplodon mirus*	Ferreira et al. [18]
**Species that have undergone a change in their taxonomic entity**		
Eulipotyphla/Soricidae	*Neomys anomalus*	Igea et al. [19]
Chiroptera/Vespertilionidae	*Myotis escalerai*	ICNF [13]
Rodentia/Cricetidae	*Microtus rozianus*	Paupério et al. [20]
**Vagrant species**		
Cetacea/Balaenopteridae	*Balaenoptera edeni*	Ferreira et al. [18]
Cetacea/Delphinidae	*Globicephala macrorhynchus*	Ferreira et al. [17]
Cetacea/Delphinidae	*Lagenodelphis hosei*	Ferreira et al. [17]
Cetacea/Delphinidae	*Lagenorhynchus acutus*	Vingada and Eira [21]
Cetacea/Delphinidae	*Lagenorhynchus albirostris*	Vingada and Eira [21]
Cetacea/Physeteridae	*Kogia sima*	Sousa [22]

**Table 2:** Records and their location (within Natura 2000 Special Areas of Conservation SACs and others) obtained for non-volant small mammals (Eulipotyphla, Rodentia) and bats (Chiroptera).

New Species	Number of Records	Type of Records	Location
Non-volant Small Mammals			
*Neomys anomalus*	94	Pellets (1), Presence signs (93, incl. 46 droppings)	10 SACs +2
*Chionomys nivalis*	1	Presence signs (1)	1 SAC
*Microtus rozianus*	305	Pellets (161), Hairs (5), Presence signs (139, incl. 40 droppings)	12 SACs +3
Bats			
*Eptesicus isabellinus*	12	Captures (12)	5 SACs
*Myotis escalerai*	88	Captures (86), acoustic detections (2)	15 SACs +7

**Table 3:** Genetic identification of non-volant small mammal and bat species.

Taxonomic Group	Mitochondrial Marker	Nr. Analyzed Samples		Genetic ID	Samples Correctly Identified
		Per Marker	Total		
Small Mammals	Cyt B	**65**	238	198 (83.2%)	*N.anomalus*—30 *M. rozianus*—28
	12 S	173			
Bats	COI	448	448	430 (96%)	*E.isabellinus*—10 *M. escalerai*—14

**Table 4:** Compiled records and type of occurrence of cetaceans off mainland Portugal, including vagrant species (adapted from [21,49,50]).

Species	Number Records	Type of Records			
		Strandings	Location Strandings	Visual Detections	Location Sightings
*Stenella frontalis*	44	2	South coast	42	West and South coast
*Mesoplodon bidens*	3	3	West coast	-	-
*Mesoplodon mirus*	6	6	West and South coast	-	-
*Balaenoptera edeni*	3	1	West coast	2	West and South coast
*Globicephala macrorhynchus*	6	3	West coast	3	West coast
*Lagenodelphis hosei*	2	2	West coast	-	-
*Lagenorhynchus acutus*	1	-	-	1	West coast
*Lagenorhynchus albirostris*	6	-	-	6	West coast
*Kogia sima*	2	2	South coast	-	-

**Table 5:** New species (except vagrant species), occurrence, population trend, and IUCN categories (abbreviations as in main text; adapted from [9]).

New Species	Occurrence in the Country			Population Trend		IUCN Category	
	Extent of Occurrence	Area of Occupancy	Range	Portugal	Global	Portugal	Global
Eulipotyphla							
*Neomys anomalus*	˃20,000 km^2^	˂500 km^2^	Northern area	Decreasing	-	VU	-
Chiroptera							
*Eptesicus isabellinus*	˃20,000 km^2^	˂500 km^2^	Throughout the country	Unknown	Unknown	LC	LC
*Myotis crypticus*	-	-	Northern area (one location)	Unknown	Decreasing	NE	NT
*Myotis alcathoe*	-	-	Northwest (one location)	Unknown	Unknown	NE	DD
*Myotis escalerai*	˃10,000 km^2^	˂2000 km^2^	Throughout the country	Unknown	Decreasing	VU	NT
Rodentia							
*Chionomys nivalis*	˂100 km^2^	~10 km^2^	Northeast (one location)	Unknown	Stable	DD	LC
*Microtus rozianus*	˃20,000 km^2^	˂500 km^2^	Northern and central areas	Decreasing	-	VU	-
Cetacea							
*Stenella frontalis*	-	-	Off the coast	Unknown	Unknown	DD	LC
*Mesoplodon mirus*	-	-	Unknown	Unknown	Unknown	DD	LC
*Mesoplodon bidens*	-	-	Unknown	Unknown	Unknown	DD	LC

## Data Availability

The original contributions presented in this study are contained within the article or Supplementary Materials. The generated sequence data have been submitted to the European Nucleotide Archive and are available under project ENA—PRJEB79154. More detailed information on the data presented in this study is available upon request from the corresponding author.

## Supplementary Materials

The following supporting information can be downloaded at: https://www.mdpi.com/article/10.3390/ani14172514/s1, S1: Project Description, Figure S1. Map of mainland Portugal, indicating the Special Areas of Conservation (SACs); S2—Geographic information on the newly documented small mammal and bat species, Table S1. Locations of new occurrence records within SACs; S3—Geographic information of strandings of newly cetacean species, Table S2. Compiled occurrence data records of standings of newly cetacean species, including vagrant species.
