# Supplementary material for: New Additions to the Mammal List Documented in the Portuguese Red Data Book
Source: Animals (Basel). 2024 Aug 29;14(17):2514. doi: 10.3390/ani14172514 (PMC11393906; doi:10.3390/ani14172514)
Supplement: Supplementary file 1 [file animals-14-02514-s001.zip › animals-3158254-supplementary.pdf]

Supplementary Materials

S1 – Project Description

Title: Revision of the Red Book of Mammals of Mainland Portugal and Contribution to the Assessment of its Conservation Status.

Institutional partners (Teams): Faculty of Science of Lisbon (Maria da Luz Mathias, project coordinator; Clara Grilo, executive coordinator; Joaquim Tapisso, Miguel Rosalino, Ana Rainho, Margarida Santos-Reis and Jorge Palmeirim, members of the technical-scientific committee), University of Trás-os-Montes e Alto Douro (João Alexandre Cabral, team coordinator), University of Aveiro (Carlos Fonseca, team coordinator; Nuno Negrões, member of the technical-scientific committee), Portuguese Wildlife Society (Marisa Ferreira, team coordinator), University of Porto (Paulo C. Alves, team coordinator; Joana Paupério, member of the technical-scientific committee), University of Évora (António Mira, team coordinator; Ricardo Pita, member of the technical-scientific committee), Institute for Nature Conservation and Forests (Maria João Cabral, Mário Reis, Margarida Lopes-Fernandes and Luisa Rodrigues, members of technical-scientific committee)

Geographic coverage: Mainland Portugal (S1.1)

Main temporal coverage: 2005-2021

Project duration: 2019-2021

Design description: For small mammals, eulipotyphlans and rodents, records were primarily obtained through owl pellets and hairs analyses, and the detection of traces. Bats were captured using nests at selected survey points, and acoustic stations positioned around these points facilitated their detection. Traditional genetic methods were used to confirm identification of small mammals and bats species, in cases of uncertain morphological identification. Most marine mammal records were compiled from visual observations and stranded deceased animals, supplemented by records from the National Strandings Database.

Funding: Main funding came from the Operational Program Sustainability and Efficiency in the Use of Resources, Ref: POSEUR-03-2215-FC-000097. Additional funding came from the Fundo Ambiental (Ministry of Environment and Climatic Action) and the academic partners.

Management entities: Association for the Research and Development of Science (FCiências.ID) and Institute for Nature Conservation and Forests.

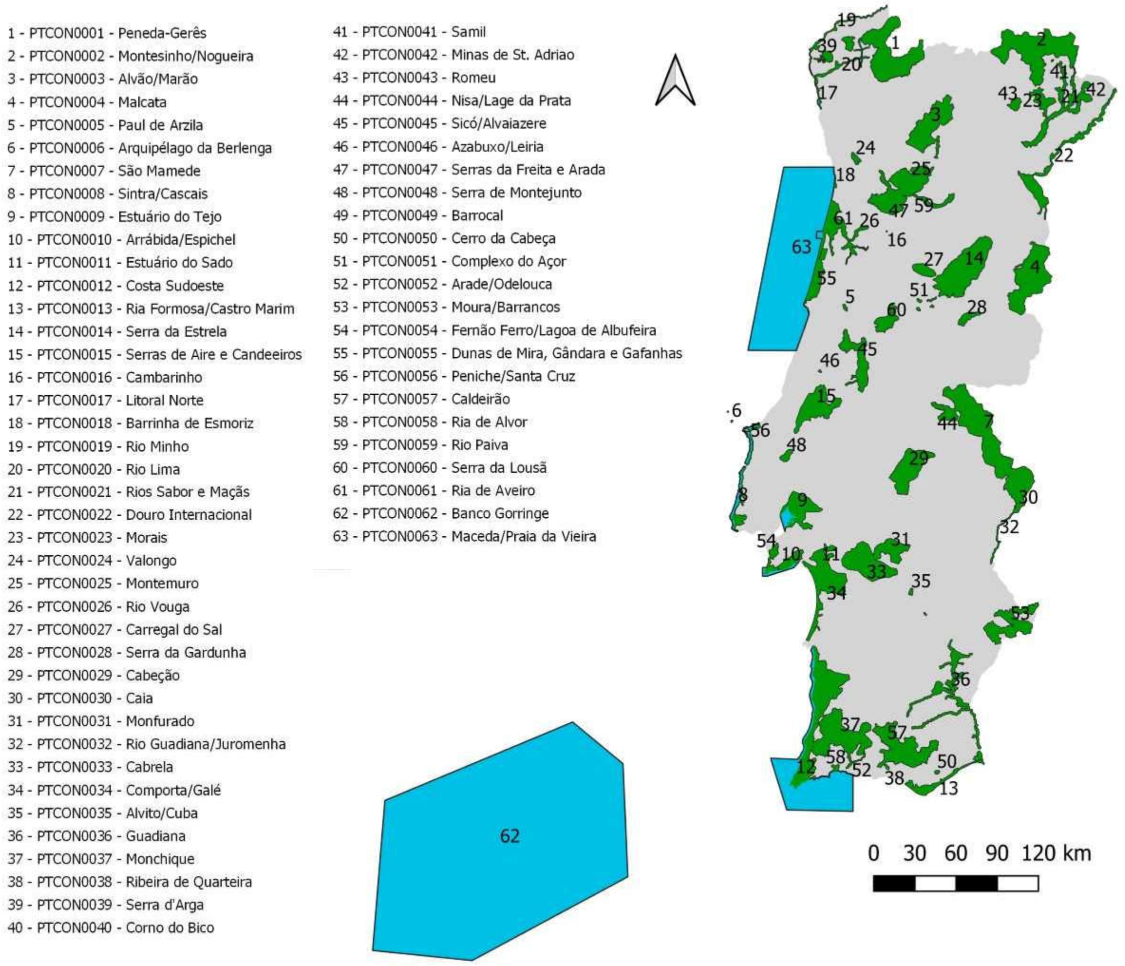

Figure S1. Map of mainland Portugal, indicating the Special Areas of Conservation (SACs) within Nature 2000 Network

S2 – New geographic information on the newly documented small mammal and bat species

Table S1. Locations of new occurrence records within Natura 2000 Network (SACs); geographic coordinates correspond to the location of a sole capture, or the one with the highest number of records; for locations outside the SACs, the coordinates pertain to the nearest locality where the species were recorded.

Location	Country Code	Geographical coordinates		Species
SACs		Longitude	Latitude	
Peneda-Gerês	PTCON0001	-8.19778	42.03582	*Myotis escalerai, Microtus rozianus, Neomys anomalus*

Montesinho/Nogueira	PTCON0002	-7.01821804	41.91862075	*Microtus rozianus, Neomys anomalus Chionomys nivalis*
Alvão-Marão	PTCON0003	-7.93683	41.34987	*Myotis escalerai, Microtus rozianus, Neomys anomalus*
Malcata	PTCON0004	-7.09644	40.185921	*Myotis escalerai, Neomys anomalus*
São Mamede	PTCON0007	-7.44953	39.40010	*Eptesicus isabellinus, Myotis escalerai*
Sintra-Cascais	PTCON0008	-9.42155	38.79326	*Myotis escalerai*
Serra da Estrela	PTCON0014	-7.30956	40.61407	*Neomys anomalus*
Litoral Norte	PTCON0017	-8.7975778	41.8740275	*Microtus rozianus*
Rio Minho	PTCON0019	-8.6492953	41.945071	*Microtus rozianus*
Rio Lima	PTCON0020	-8.6393188	41.766463	*Microtus rozianus, Neomys anomalus*
Douro Internacional	PTCON0022	-6.49738	41.33648	*Microtus rozianus*
Morais	PTCON0023	-6.90706	41.53241	*Neomys anomalus*
Valongo	PTCON0024	-8.49603	41.18131	*Myotis escalerai*
Serra Montemuro	PTCON0025	-7.87876	41.02716	*Myotis escalerai, Microtus rozianus, Neomys anomalus*
Serra da Gardunha	PTCON0028	-7.53507	40.14499	*Microtus rozianus*
Comporta/Galé	PTCON0034	-8.54951	38.33127	*Myotis escalerai*
Guadiana	PTCON0036	-7.710476	37.498054	*Eptesicus isabellinus*
Serra Arga	PTCON0039	-8.79459	41.7995	*Myotis escalerai, Microtus rozianus, Neomys anomalus*
Corno do Bico	PTCON0040	-8.56343	41.93082	*Myotis escalerai, Microtus rozianus, Neomys anomalus*
Minas St.Adrião	PTCON0042	-6.47636	41.53478	*Myotis escalerai, Microtus rozianus*
Serra Montejunto	PTCON0048	-9.00665	39.21831	*Myotis escalerai*
Barrocal	PTCON0049	-8.01017	37.20942	*Eptesicus isabellinus*
Complexo do Açor	PTCON0051	-7.71997	40.21648	*Myotis escalerai*

Moura-Barrancos	PTCON0053	-7.32038	38.10896	*Eptesicus isabellinus*
Caldeirão	PTCON0057	-7.88175	37.18105	*Eptesicus isabellinus*
Rio Paiva	PTCON0059	-7.94805	40.89821	*Myotis escalerai*
Ria de Aveiro	PTCON0061	-8.570854	40.72062	*Myotis escalerai*
Other locations				
Vale do Tua		-7.43136	41.37806	*Myotis escalerai*
Sanguinhedo		-7.65567	41.33097	*Myotis escalerai*
Gondiães		-7.84371	41.58026	*Myotis escalerai*
Caramulo		-8.16614	40.58318	*Myotis escalerai*
Nossa Sra. Boa Fé		-8.53459	41.51095	*Myotis escalerai*
Paul da Tornada		-9.13374	39.44886	*Myotis escalerai*
Mafra		-9.29069	38.95500	*Myotis escalerai*
Mondim de Basto		-8.10422;	41.36392	*Microtus rozianus*
Agilde		-8.10428	41.36379	*Microtus rozianus*
Vila de Prado		-8.481673	41.6045392	*Microtus rozianus*
Carrazeda de Ansiães		-7.32904	41.21108	*Neomys anomalus*
Marzagão		-7.33004	41.21109	*Neomys anomalus*

S3 – Geographic information of strandings of newly cetacean species

Table S2. Compiled occurrence data records of standings of newly cetacean species, including vagrant species, within Natura 2000 Network (SACs); geographic coordinates correspond to the location of a sole capture, or the one with the highest number of records; for locations outside the SACs, the coordinates pertain to the nearest locality where the species were recorded.

Location	Country Code	Geographical coordinates		Species
SACs		Longitude	Latitude	
Costa Sudoeste	PTCON0012	-8,807593	37,419685	*Mesoplodon bidens, M. mirus* (2), *Stenella frontalis, Kogia sima*
Ria Formosa, Castro Marim	PTCON0013	-8,672278	37,082246	*Mesoplodon mirus* (2), *Kogia sima,*<br>*Stenella frontalis*
Comporta, Galé	PTCON0034	-8,776767	38,204727	*Mesoplodon bidens*
Dunas de Mira	PTCON0055	-8.650607	41.0609498	*Mesoplodon mirus*
Maceda, Praia da Vieira	PTCON0063	-8,909337	40,01383	*Globicephala macrorhynchus* (2), *Lagenodelphis hosei* (2), *Mesoplodon bidens*
Other locations				
Nazaré		-9,0844909	39,609158	*Balaenoptera edeni*
Óbidos		-9,278064	39,39948	*Globicephala macrorhynchus*
Albufeira		-8,807593	37,128300	*Mesoplodon mirus*
